# Cutaneous Adverse Events With Semaglutide: A Systematic Review and Meta-Analysis of Randomized Controlled Trials

**DOI:** 10.7759/cureus.117270

**Published:** 2026-09-30

**Authors:** Samyak Ganjre, Liza Borah, Archit Khardenavis

**Affiliations:** 1 Dermatology, Raipur Institute of Medical Sciences, Raipur, IND; 2 Community Medicine, Ram Krishna Medical College Hospital and Research Centre, Bhopal, IND

**Keywords:** alopecia, cutaneous adverse events, glucagon-like peptide-1 receptor agonists (glp-1s), injection-site reactions, meta-analysis, randomized controlled trials, semaglutide, systematic review

## Abstract

Semaglutide is widely used for metabolic disease, but cutaneous adverse events are inconsistently reported. This systematic review and meta-analysis evaluated randomized comparisons of semaglutide with placebo, usual care, or non-semaglutide active treatment. The protocol was registered in the International Prospective Register of Systematic Reviews (PROSPERO; CRD420261464289). PubMed, Europe PubMed Central, trial registers, sponsor reports, and regulatory sources were examined through September 9, 2026; source verification was completed on September 21, 2026. Meta-analysis required directly available, source-verified participant event counts and denominators in both groups. Reported alopecia or hair loss and source-defined participant-level injection-site reactions were analyzed separately using common-effect Mantel-Haenszel odds ratios (ORs) without continuity correction. Risk of bias and evidence certainty were assessed for the primary syntheses. Thirty-one trials contributed verified outcome data. Twenty trials formed the primary placebo/common-control evidence set, of which 19 contributed relative-effect estimates and one reported zero injection-site reactions in both groups. Eleven additional trials provided active-comparator or design-specific evidence. Across nine comparisons, alopecia or hair loss was reported in 191/11,564 semaglutide recipients and 36/9,799 comparators (OR: 2.92, 95% confidence interval (CI): 1.99-4.27; p < 0.001; low-certainty evidence). The association persisted after exclusion of the largest cardiovascular outcomes trial with restricted ascertainment; random-effects analyses supported the primary interpretations. Eleven informative comparisons reported injection-site reactions in 156/5,066 semaglutide recipients and 114/3,483 comparators (OR: 0.86, 95% CI: 0.67-1.11; p = 0.256; very-low-certainty evidence). Active comparators and design-specific results were described separately. Semaglutide may increase reports of alopecia or hair loss; its comparative effect on injection-site reactions remains uncertain. Available-count synthesis does not resolve selective non-reporting or incomplete event ascertainment. The findings do not establish a hair-loss mechanism or injection-site safety equivalence.

## Introduction and background

Semaglutide is a glucagon-like peptide-1 (GLP-1) receptor agonist used in type 2 diabetes and chronic weight management [1,2]. The expanding use of semaglutide has increased the importance of characterizing cutaneous adverse events that may affect treatment tolerability and patients’ experience. Reported concerns include hair loss, injection-site reactions, hypersensitivity, and altered skin sensation. Regulatory information recognizes these events, although their reported frequencies vary with formulation, dose, population, and the methods used to collect adverse-event data [1,2]. Hair loss has been reported more frequently with greater weight loss, although a direct drug-mediated mechanism has not been established [2].

Previous reviews have examined dermatologic reactions across GLP-1 receptor agonists, while pharmacovigilance and observational studies have investigated associations with hair loss [3-5]. Spontaneous reports can identify potential safety signals but cannot reliably establish incidence, and observational comparisons remain susceptible to differences in treatment selection and baseline risk. Randomized trials provide complementary evidence for estimating comparative effects when adverse-event reporting is sufficiently detailed. A semaglutide-specific synthesis can therefore help clarify the available evidence while accounting for differences in outcome definitions and reporting.

Reliable synthesis requires a clear distinction between unreported outcomes and explicitly reported zero events. Frequency thresholds in safety tables can obscure uncommon events, while summing separate local-reaction terms may count the same participant more than once. These reporting characteristics are particularly relevant when evaluating reported alopecia or hair loss and composite injection-site reactions. We therefore conducted a systematic review and meta-analysis of randomized controlled trials to evaluate cutaneous adverse events associated with semaglutide, focusing on reported alopecia or hair loss and participant-level composite injection-site reactions. The analyses distinguished reported events, explicit zeros, and unavailable results to support a cautious assessment of comparative risk.

## Review

Methodology

Protocol and Eligibility

The protocol was registered in the International Prospective Register of Systematic Reviews (PROSPERO) on July 29, 2026 (CRD420261464289) [6]. Reporting was informed by the Preferred Reporting Items for Systematic Reviews and Meta-Analyses (PRISMA) 2020 statement and its harms extension [7,8]. Eligible randomized trials compared semaglutide with concurrent placebo, usual care, or non-semaglutide active treatment, with balanced co-interventions. Eligibility covered all ages, indications, languages, doses, and formulations. The registered any-route criterion was applied; injection-site analyses concerned subcutaneous treatment. Nonrandomized studies, semaglutide-only dose or formulation comparisons, and non-isolatable combinations were excluded. Crossover trials required usable first-period or appropriately paired data. Companion reports were linked to their trial, and eligible trials remained in the review inventory regardless of cutaneous outcome availability.

Information Sources and Selection

PubMed and Europe PubMed Central were searched from inception through September 9, 2026. Searches combined semaglutide, its development identifiers (NN9535, NN-9535, NN 9535, NN9924, NN-9924, NN 9924, NNC 0113-0217, and NNC-0113-0217), and brand names (Ozempic, Rybelsus, and Wegovy) with trial terms: randomized controlled trial, controlled clinical trial, clinical trial, random*, placebo*, trial, double blind, single blind, and groups. No language, publication date, geographic, or cutaneous outcome restriction was applied. Animal-only records were excluded by the PubMed query or during screening. The registered searches of Embase, the Cochrane Central Register of Controlled Trials (CENTRAL), Scopus, and Web of Science were not completed because usable database access or search results could not be obtained. The dated access records documented access errors for Embase and CENTRAL, a timeout for Scopus, and a Web of Science interface without usable search results. Europe PubMed Central was searched as an additional source. Trial register, sponsor, regulatory, and reference list searches broadened coverage but were not considered equivalent substitutes for the omitted databases.

Additional sources comprised ClinicalTrials.gov, the European Union Clinical Trials Register, the World Health Organization International Clinical Trials Registry Platform, the public Novo Nordisk results index, United States Food and Drug Administration and European Medicines Agency documents, and reference lists. Register and sponsor searches used semaglutide, NN9535, NN9924, Ozempic, Rybelsus, and Wegovy. Duplicate bibliographic records were reconciled using PubMed identifiers, digital object identifiers, and title metadata. Initial screening removed clearly secondary, nonrandomized, and nonhuman evidence. Remaining records underwent source-based eligibility assessment using publications, abstracts, trial registrations, and companion sources. Reports were linked to trials using registration identifiers, sponsor study codes, and study characteristics; multiple reports of one trial were not counted as separate studies. All three authors independently performed record screening, eligibility assessment, data extraction, Cochrane Risk of Bias 2 (RoB 2) assessments, Risk Of Bias due to Missing Evidence (ROB-ME)-informed assessments of missing evidence, and Grading of Recommendations Assessment, Development and Evaluation (GRADE) assessments. Disagreements at any stage were resolved through mutual discussion until consensus was reached.

The exact database strategies are provided below. Field tags specify Medical Subject Headings (Mesh, mh, or MESH), title and abstract (tiab or TITLE_ABS), or publication type (pt or PUB_TYPE); an asterisk denotes truncation.

PubMed search: In PubMed, the following search string was used: ("Semaglutide"[Mesh] OR semaglutide[tiab] OR NN9535[tiab] OR "NN-9535"[tiab] OR "NN 9535"[tiab] OR NN9924[tiab] OR "NN-9924"[tiab] OR "NN 9924"[tiab] OR "NNC 0113-0217"[tiab] OR "NNC-0113-0217"[tiab] OR Ozempic[tiab] OR Rybelsus[tiab] OR Wegovy[tiab]) AND (randomized controlled trial[pt] OR controlled clinical trial[pt] OR clinical trial[pt] OR random*[tiab] OR placebo*[tiab] OR trial[tiab] OR "double blind"[tiab] OR "single blind"[tiab] OR groups[tiab]) NOT (animals[mh] NOT humans[mh]).

Europe PubMed Central search: For Europe PubMed Central search, the following search string was used: (TITLE_ABS:semaglutide OR TITLE_ABS:NN9535 OR TITLE_ABS:"NN-9535" OR TITLE_ABS:"NN 9535" OR TITLE_ABS:NN9924 OR TITLE_ABS:"NN-9924" OR TITLE_ABS:"NN 9924" OR TITLE_ABS:"NNC 0113-0217" OR TITLE_ABS:"NNC-0113-0217" OR TITLE_ABS:Ozempic OR TITLE_ABS:Rybelsus OR TITLE_ABS:Wegovy OR MESH:"Semaglutide") AND (PUB_TYPE:"randomized controlled trial" OR PUB_TYPE:"controlled clinical trial" OR PUB_TYPE:"clinical trial" OR TITLE_ABS:random* OR TITLE_ABS:placebo* OR TITLE_ABS:trial OR TITLE_ABS:"double blind" OR TITLE_ABS:"single blind" OR TITLE_ABS:groups).

Study Selection and Final Analytical Sample

Study eligibility was distinguished from eligibility for an individual synthesis. Publications, abstracts, registrations, and companion reports were linked using registration identifiers, sponsor study codes, and trial characteristics. Multiple reports of a trial were treated as complementary sources rather than independent studies. The review inventory retained eligible trial designs irrespective of whether the two primary outcomes were reported. Source access was recorded separately from trial-level eligibility; assessment used the available published documents, abstracts, and linked trial sources.

The final verified outcome-data set comprised 31 distinct randomized trials. Twenty trials provided primary placebo/common-control evidence and were represented by 18 principal trial articles and two additional registry results reports. Nine trials provided alopecia data, and 12 provided injection-site-reaction data, with one trial contributing to both outcomes. One injection-site trial reported zero events in both groups and was retained descriptively; therefore, 19 distinct trials contributed to the primary relative-effect meta-analyses. Nine additional active-comparator trials and two additional design-specific trials contributed separate descriptive evidence. Principal articles, all supporting reports, and distinct trials were counted separately.

Bibliographic searches covered records available through September 9, 2026. Eligible comparisons were cross-checked against trial registrations and available supporting documents, with final source verification completed on September 21, 2026.

Outcomes and Data Handling

The primary outcomes were the numbers of participants with at least one treatment-emergent report of alopecia or hair loss and with at least one investigator- or protocol-defined injection-site reaction. The longest usable randomized on-treatment period or follow-up was selected. For injection-site reactions, the source had to report a participant-level category or composite; individual local-reaction terms were not summed. An isolated Medical Dictionary for Regulatory Activities preferred term was not assumed to represent all injection-site reactions. Serious and nonserious event rows were kept distinct when participant overlap was unknown. Clinically comparable semaglutide arms were combined, with each shared control used once. Randomized, safety, and exposed denominators were distinguished.

Quantitative synthesis was restricted to directly available, source-verified event counts and denominators for both groups. This post hoc restriction differed from the registered allowance for unambiguous reconstruction. Reconstructed counts, unverified numerators, unavailable arm data, and results outside an identifiable randomized comparison were excluded from pooling. Explicit zeros were retained; outcomes absent from thresholded tables were classified as unreported. Exact outcome tables from public sources were eligible regardless of full-article access. Pooling used observed counts only. Completeness of adverse-event ascertainment was considered separately from completeness of the extracted counts.

Risk of Bias and Certainty

Outcome-level assessments for the primary placebo/common-control evidence used the Cochrane Risk of Bias 2 (RoB 2) tool for the effect of assignment to intervention [9]. The five domains addressed randomization, deviations from intended intervention, missing outcome data, outcome measurement, and selection of the reported result. Protocols, analysis plans, registrations, and reports informed these assessments. Cross-source comparisons were informed by the Risk Of Bias due to Missing Evidence (ROB-ME) framework [10]. The Grading of Recommendations Assessment, Development and Evaluation (GRADE) approach assessed the primary pooled bodies of evidence for risk of bias, inconsistency, indirectness, imprecision, and publication bias, starting randomized evidence at high certainty [11]. Assessments considered the reported observation windows and adverse-event collection rules. Unavailable eligible outcome results informed the GRADE publication/non-reporting-bias domain, whereas limitations in included results informed risk of bias. Restricted adverse-event ascertainment in the Semaglutide Effects on Cardiovascular Outcomes in People with Overweight or Obesity (SELECT) trial was considered only under risk of bias and was not counted again as missing evidence.

Statistical Analysis

The common-effect Mantel-Haenszel odds ratio (OR) model was retained as the primary sparse-event analysis because it combines stratified arm-level counts without an arbitrary continuity correction and accommodates unequal allocation ratios [12,13]. Its use does not establish a common biological effect across indications, formulations, doses, or observation periods. Clinical comparability was limited to the defined outcome and comparator class, and active-comparator and design-specific results were considered separately. Nonsignificant homogeneity tests were not interpreted as evidence that treatment effects were identical. The Robins-Breslow-Greenland variance provided 95% confidence intervals (CIs); two-sided Wald z tests assessed log(OR) = 0 [14]. Individual-study ORs and equal-tailed exact 95% CIs were obtained from the conditional noncentral hypergeometric distribution. Double-zero comparisons were retained descriptively. Active comparators were evaluated separately according to their regimens, devices, and injection exposure.

Homogeneity of ORs was assessed using the Tarone-adjusted Breslow-Day chi-square test with k - 1 degrees of freedom, where k is the number of informative comparisons [15]. Interpretation accounted for sparse expected event cells. Leave-one-out analyses examined individual-trial influence. Exploratory sensitivity analyses excluded the cardiovascular outcomes trial reported by Lincoff et al. [16] for restricted ascertainment and, separately, the weight-management trial reported by Wilding et al. [17] and the alcohol-use trial reported by Klausen et al. [18] for their reliance on indexed primary-table text rather than retrieved full articles. The registered sensitivity restriction excluding phase I or registry-only evidence was applied to the additional informative injection-site comparison; a combined exclusion analysis was also performed. Absolute effects were calculated from the pooled OR and the observed pooled comparator risk in informative comparisons. Double-zero comparisons were excluded from relative-effect calculations and their associated participant totals but retained descriptively. The common-effect model and these sensitivity analyses represented a narrower synthesis than the registered plan. Sparse events and variable ascertainment limited the usefulness of funnel-plot regression and prediction intervals. All tests were two-sided, without multiplicity adjustment.

To examine sensitivity to the common-effect assumption, an exploratory conditional hypergeometric-normal random-effects model was fitted to the same informative comparisons. Trial-specific log odds ratios (ORs) were assumed to follow a normal distribution with mean μ and variance τ². Conditioning on each trial’s total event count eliminated its baseline odds; maximum likelihood estimation used numerical integration without continuity correction. Single-zero comparisons were retained, and double-zero comparisons contributed no conditional relative-effect information. Confidence intervals for exp(μ) were obtained by profiling over τ; two-sided likelihood-ratio tests assessed μ = 0. A variance estimate at the boundary τ² = 0 was not interpreted as proof of no heterogeneity.

Software

Analyses used Python 3.12.14 (Python Software Foundation, Beaverton, OR), NumPy 2.3.5 (NumPy Developers), SciPy 1.17.0 (SciPy Developers), and statsmodels 0.15.0 (https://www.statsmodels.org/). Figures used Matplotlib 3.10.8 (Matplotlib development team). NumPy, SciPy, and Matplotlib receive fiscal and administrative support from NumFOCUS, 6500 River Place Blvd, Bldg 7-250, Austin, TX 78730, USA. Direct formula calculations were cross-checked against statsmodels.

Results

Study Selection and Characteristics

The database searches identified 6,905 records: 2,066 from PubMed and 4,839 from Europe PubMed Central. Removal of 2,073 duplicates left 4,832 records for screening. Initial screening excluded 1,700 records, and the remaining 3,132 underwent source-based eligibility assessment. A further 2,670 records were excluded, and two had indeterminate designs. The 460 retained bibliographic sources comprised 406 empirical trial reports or companions, 50 protocol or design companions, and four ancillary sources. Complete published documents were retrieved for 238 empirical reports; 160 were available as abstracts and eight through metadata or companion sources. The 238 complete documents comprised 214 research articles or companions, one preprint, three corrections, and 20 conference abstracts.

ClinicalTrials.gov searches yielded 1,061 entries; removal of 281 repeated entries left 780 unique records. Assessment identified 353 eligible trial designs, 425 excluded designs, and two indeterminate designs. Additional searches assessed 97 unique European Union Clinical Trials Register records, eight World Health Organization International Clinical Trials Registry Platform records, 101 Novo Nordisk results synopses, and five reports identified through citation searching. These sources overlapped and were linked at trial level.

Cross-source linkage yielded an inventory of 378 eligible unique trial designs, including 162 with an empirical results source and 216 without an identified results source. Thirty-one distinct trials contributed verified participant-level data for at least one of the two primary outcomes. The primary placebo/common-control evidence comprised 20 trials, represented by 18 principal trial articles [16-33] and two registry results reports [34,35]. Nine trials provided alopecia data and 12 provided injection-site-reaction data. One trial in people with human immunodeficiency virus (HIV)-associated lipohypertrophy contributed to both outcomes. The kidney-transplant-candidacy trial reported zero injection-site reactions in both groups and was retained descriptively [35]. Thus, 19 distinct trials contributed to the primary relative-effect meta-analyses: nine alopecia comparisons and 11 injection-site comparisons. Participants in these primary analyses were adults receiving treatment for weight management, type 2 diabetes, cardiovascular risk, HIV-associated lipohypertrophy, or alcohol use disorder.

Active-comparator evidence involved 10 trials, one of which also contributed to a primary synthesis. A randomized-withdrawal comparison and a regional double-zero comparison came from two further trials. These comparisons therefore added 11 distinct trials to the 20-trial primary evidence set. The remaining 131 empirically reported eligible trials were retained in the source inventory outside the verified primary-outcome dataset; they were not counted as design exclusions.

Outcome-specific exclusions under the direct-count rule comprised reconstructed alopecia counts from the randomized-withdrawal and adolescent weight-management trials (ClinicalTrials.gov identifiers NCT03548987 and NCT04102189), reconstructed injection-site counts from the basal-insulin add-on trial (NCT02305381) and the phase 2 nonalcoholic steatohepatitis trial, and injection-site numerators lacking direct source verification from the trial by O’Neil et al. [21] and the semaglutide monotherapy comparison in NCT05567796. Verified alopecia counts from O’Neil’s trial remained eligible. The additional informative registry comparison reported investigator-defined injection-site reactions in 5/48 versus 0/48 participants [34]; the additional double-zero trial reported 0/7 versus 0/8 [35]. These directly reported participant counts met the registered outcome and source criteria. Figure 1 distinguishes the source inventory, the 31 trials contributing verified outcome data, and the 19 trials contributing primary relative-effect estimates.

**Figure 1:**
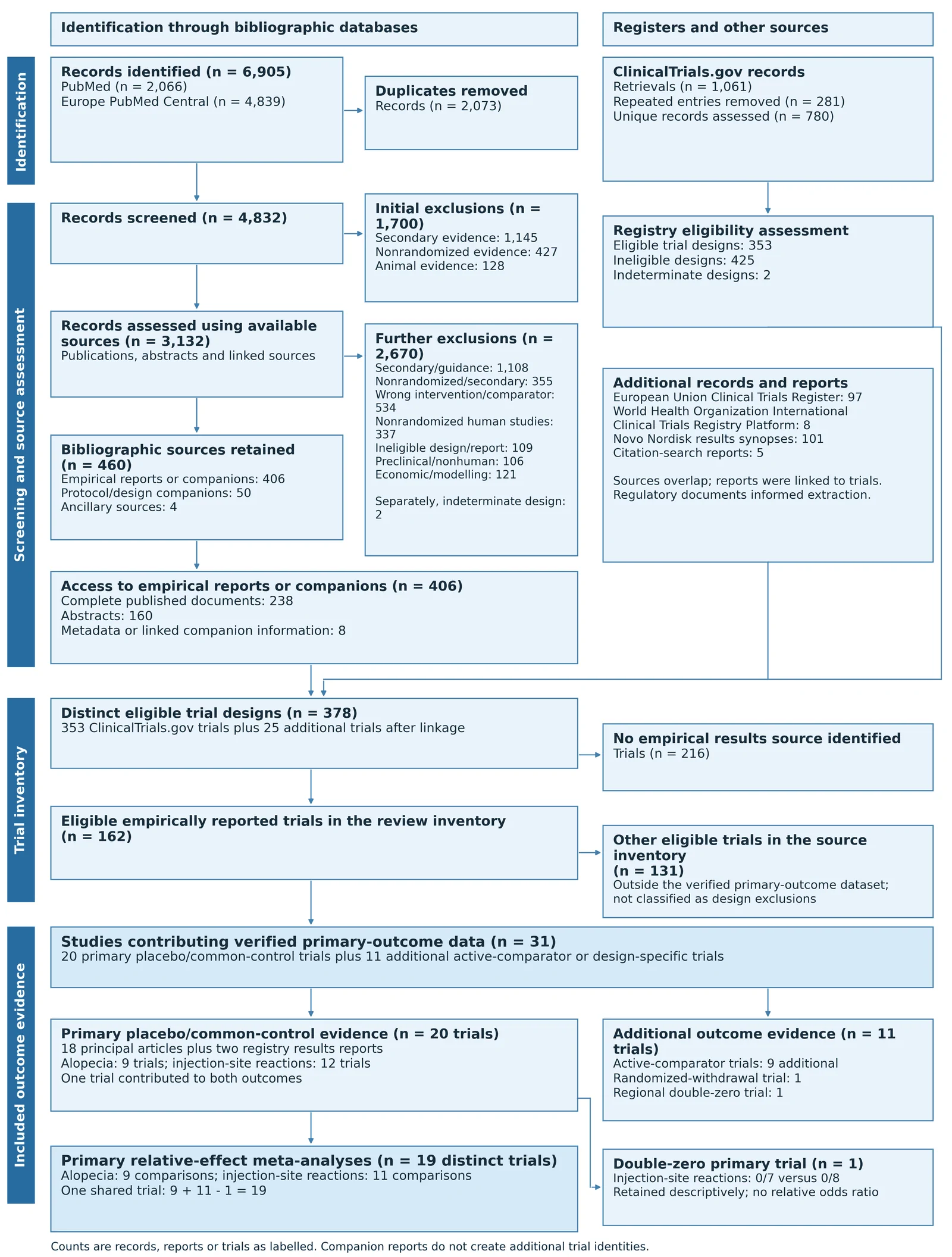
Study identification, source assessment, and inclusion in the primary meta-analyses Adapted from the PRISMA 2020 flow diagram [7] under the Creative Commons Attribution 4.0 license. Counts distinguish bibliographic records, reports, and distinct trial designs. Source-based assessment used publications, abstracts, registrations, and linked companion reports; the 3,132 records were not a count of retrieved full-text articles. The 406-report access breakdown describes retained empirical reports or companions. Complete published documents included 214 research articles or companions, one preprint, three corrections, and 20 conference abstracts. Register, sponsor, citation, and regulatory sources overlap and were linked before trial-level counting. Of 162 eligible trials with empirical results sources, 31 contributed verified primary-outcome data: 20 primary placebo/common-control trials, nine additional active-comparator trials, and two additional design-specific trials. The other 131 remained in the source inventory. The 20 primary trials were represented by 18 principal articles and two registry results reports; one reported zero injection-site reactions in both groups. Nineteen distinct trials contributed to the relative-effect meta-analysis. Counts of principal reports do not include all supporting companion sources. PRISMA: Preferred Reporting Items for Systematic Reviews and Meta-Analyses

The characteristics of the 31 randomized trials contributing outcome data are summarized in Table 1. Twenty trials contributed primary placebo/common-control evidence, and 11 additional trials contributed active-comparator or design-specific evidence [16-43].

**Table 1:** Characteristics of randomized trials contributing cutaneous outcome data All studies were randomized trials in adults. In each row, n indicates the numbers of semaglutide/comparator participants in the reviewed outcome comparison; the additional liraglutide group in the trial by O’Neil et al. [21] is shown separately. Arm sizes are outcome-analysis denominators and may differ from total trial enrollment. Doses indicate target or maintenance regimens after titration. Treatment duration and longer source-reported safety windows are distinguished; a registry-wide window need not equal the on-treatment window of an individual adverse-event table. The Yale regimen is described without a target dose because the registry and protocol specify different targets. The kidney-transplant trial reported zero injection-site reactions in both groups and was retained descriptively. The O’Neil trial is listed once despite contributing placebo and active-comparator evidence. NCT identifiers denote ClinicalTrials.gov registration numbers. Sources are cited in each row [16-43]. This is a descriptive table; no hypothesis test applies. mg: milligram

Study and analyzed participants	Population	Semaglutide regimen	Comparator	Treatment and safety observation
Wilding et al. (2021) [17], NCT03548935, n = 1,306/655	Overweight or obesity without diabetes	Subcutaneous 2.4 mg weekly	Matched placebo; lifestyle advice in both groups	68 weeks; registry safety window through week 75
Garvey et al. (2022) [19], NCT03693430, n = 152/152	Overweight or obesity without diabetes	Subcutaneous 2.4 mg weekly	Matched placebo; lifestyle advice in both groups	104 weeks; safety through week 111
Kadowaki et al. (2022) [20], NCT03811574, n = 299/101	East Asian adults with overweight or obesity, with or without type 2 diabetes	Subcutaneous 1.7 or 2.4 mg weekly	Dose-matched placebo; lifestyle advice in both groups	68 weeks; registry safety window through week 75
O’Neil et al. (2018) [21], NCT02453711, n = 718/136, liraglutide n = 103	Obesity without diabetes	Subcutaneous 0.05-0.4 mg daily; seven regimens combined	Pooled placebo; liraglutide 3 mg daily considered separately	52 weeks; safety through week 59
Knop et al. (2023) [22], NCT05035095, n = 334/333	Overweight or obesity without diabetes	Oral 50 mg daily	Matched oral placebo; lifestyle advice in both groups	68 weeks; safety through week 75
Wharton et al. (2025) (oral) [23], NCT05564117, n = 204/102	Overweight or obesity without diabetes	Oral 25 mg daily	Matched oral placebo; lifestyle advice in both groups	64 weeks; safety through week 71
Wharton et al. (2025) (injection) [24], NCT05646706, n = 1,205/201	Obesity without diabetes	Subcutaneous 2.4 or 7.2 mg weekly	Matched placebo	72 weeks; safety through week 81
Lincoff et al. (2023) [16], NCT03574597, n = 8,803/8,801	Overweight or obesity, established cardiovascular disease, without diabetes	Subcutaneous 2.4 mg weekly	Matched placebo; standard care in both groups	Median treatment exposure: 37.3 months semaglutide, 38.6 months placebo
Lim et al. (2025) [25], NCT04998136, n = 101/49	Thai and South Korean adults with obesity	Subcutaneous 2.4 mg weekly	Matched placebo; lifestyle advice in both groups	44 weeks; safety through week 49
Amin et al. (2025) [26], NCT05579977, n = 73/75	Type 2 diabetes	Oral 14 mg daily	Placebo within the diabetes stratum	Trial stopped early; reported safety observation up to week 28
Eckard et al. (2024) [27], NCT04019197, n = 54/54	Human immunodeficiency virus infection with lipohypertrophy, without diabetes	Subcutaneous 1.0 mg weekly	Matched placebo	32 weeks
Tronieri et al. (2026) [28], NCT05548647, n = 72/48	Overweight or obesity	Subcutaneous 2.4 mg weekly	Matched placebo; behavioral treatment in both groups	Initial randomized period: weeks 0-60
Sorli et al. (2017) [29], NCT02054897, n = 258/129	Treatment-naive type 2 diabetes	Subcutaneous 0.5 or 1.0 mg weekly	Matched placebo	30 weeks; safety follow-up through last dose + 42 days
Marso et al. (2016) [30], NCT01720446, n = 1,648/1,649	Type 2 diabetes with high cardiovascular risk	Subcutaneous 0.5 or 1.0 mg weekly	Dose-matched placebo; both groups received standard care	104 weeks; follow-up through week 109
Davies et al. (2021) [31], NCT03552757, n = 805/402	Overweight or obesity with type 2 diabetes	Subcutaneous 1.0 or 2.4 mg weekly	Matched placebo; lifestyle advice in both groups	68 weeks; registry safety window through week 75
Wadden et al. (2021) [32], NCT03611582, n = 407/204	Overweight or obesity without diabetes	Subcutaneous 2.4 mg weekly	Matched placebo; intensive behavioral therapy in both groups	68 weeks; registry safety window through week 75
Klausen et al. (2026) [18], NCT05895643, n = 54/54	Alcohol use disorder with obesity	Subcutaneous, titrated to 2.4 mg weekly	Weekly saline placebo	26 weeks
Stanley et al. (2026) [33], NCT05073835, n = 35/35	Suboptimal weight loss after metabolic bariatric surgery	Subcutaneous 2.4 mg weekly	Matched placebo	68 weeks; scheduled safety observation through week 74
Yale University, Yale food-cue trial (2026) [34], NCT04779697, n = 48/48	Adults with obesity	Subcutaneous, titrated weekly	Matched placebo	12 weeks
Geisinger Clinic, Kidney-transplant candidacy trial (2024) [35], NCT04741074, n = 7/8	Overweight or obesity, type 2 diabetes and chronic kidney disease limiting transplant candidacy	Subcutaneous, titrated to 1.0 mg weekly	Matched placebo	9 months
Canadian Agency for Drugs and Technologies in Health, Exenatide comparison (2019) [36], NCT01885208, n = 404/405	Type 2 diabetes receiving one or two oral antidiabetic drugs	Subcutaneous 1.0 mg weekly	Subcutaneous extended-release exenatide 2 mg weekly	56 weeks; safety through last dose + 42 days
Canadian Agency for Drugs and Technologies in Health, Sitagliptin comparison (2019) [36], NCT01930188, n = 818/407	Type 2 diabetes receiving metformin and/or a thiazolidinedione	Subcutaneous 0.5 or 1.0 mg weekly	Sitagliptin 100 mg daily with matching weekly injection	56 weeks
Canadian Agency for Drugs and Technologies in Health, Insulin glargine comparison (2019) [36], NCT02128932, n = 722/360	Insulin-naive type 2 diabetes receiving metformin with or without a sulfonylurea	Subcutaneous 0.5 or 1.0 mg weekly	Daily subcutaneous insulin glargine	30 weeks; safety through last dose + 42 days
Canadian Agency for Drugs and Technologies in Health, Dulaglutide comparison (2019) [36], NCT02648204, n = 601/598	Type 2 diabetes receiving metformin	Subcutaneous 0.5 or 1.0 mg weekly	Subcutaneous dulaglutide 0.75 or 1.5 mg weekly	40 weeks; safety follow-up for approximately 5 further weeks
Aronne et al. (2025) [37], NCT05822830, n = 376/374	Obesity or overweight with a weight-related condition, without diabetes	Subcutaneous maximum tolerated dose: 1.7 or 2.4 mg weekly	Subcutaneous tirzepatide maximum tolerated dose: 10 or 15 mg weekly	72 weeks
Novo Nordisk, Liraglutide comparison (2021) [38], NCT04074161, n = 126/127	Overweight or obesity without diabetes	Subcutaneous 2.4 mg weekly	Subcutaneous liraglutide 3 mg daily	68 weeks; registry safety window through week 75
Frías et al. (2021) [39], NCT03987919, n = 469/1,409	Type 2 diabetes receiving metformin	Subcutaneous 1.0 mg weekly	Subcutaneous tirzepatide 5, 10, or 15 mg weekly	40 weeks; safety follow-up through week 44
Novo Nordisk, Japan oral-drug comparison (2016) [40], NCT02207374, n = 480/120	Japanese adults with type 2 diabetes	Subcutaneous 0.5 or 1.0 mg weekly	Additional oral antidiabetic drug	56 weeks; safety through last dose + 42 days
Novo Nordisk, Japan sitagliptin comparison (2016) [41], NCT02254291, n = 205/103	Japanese adults with type 2 diabetes	Subcutaneous 0.5 or 1.0 mg weekly	Sitagliptin 100 mg daily	30 weeks; safety through last dose + 42 days
Novo Nordisk, Randomized withdrawal comparison (2020) [42], NCT03548987, n = 534/268	Overweight or obesity without diabetes; completed semaglutide run-in	Continue subcutaneous 2.4 mg weekly	Switch to matched placebo	48 randomized weeks after 20-week semaglutide run-in
Gu et al. (2025) (China subgroup) [43], NCT04251156, n = 195/105	Chinese adults with overweight or obesity, with or without type 2 diabetes	Subcutaneous 2.4 mg weekly	Matched placebo	44 weeks; trial safety window through week 51

Risk of Bias and Missing Results

For alopecia, the trial reported by Lincoff et al. [16] was judged at high risk of bias, the trial reported by Lim et al. [25] at low risk, and seven comparisons at some concerns. Restricted collection of nonserious adverse events in the Lincoff trial limited ascertainment despite complete reported arm counts. Other concerns involved observation windows, missing outcomes, or selection of reported analyses. The 11 informative injection-site comparisons and the additional double-zero comparison were judged at some concerns. For the two additional registry trials, incomplete follow-up remained relevant despite the explicit adverse-event denominators. Frequency thresholds and unavailable outcome-specific tables raised concerns about missing results. Combined regulatory alopecia results for three pivotal weight-management trials were excluded from pooling because unique trial-level numerators were unavailable. Unreported injection-site outcomes were treated as missing data. Table 2 summarizes the primary pooled effects and statistical tests.

**Table 2:** Primary effects using verified arm-level event counts Events/participants indicates participants with at least one event divided by the source-specific randomized, safety, or exposed denominator. Comparators were placebo or common control. Effects used a common-effect Mantel-Haenszel model without continuity correction, Robins-Breslow-Greenland variance, and two-sided Wald z tests. Tarone-adjusted Breslow-Day homogeneity tests yielded χ² = 10.95, 8 degrees of freedom, p = 0.205 for alopecia and χ² = 11.05, 10 degrees of freedom, p = 0.354 for injection-site reactions. Expected event cells were below five in 7/9 and 5/11 comparisons, respectively, limiting interpretation of these asymptotic tests. The additional injection-site comparison with 0/7 versus 0/8 participants was retained descriptively and excluded from the relative-effect totals. Unreported outcomes were treated as missing, not zero.

Outcome	Informative trials	Semaglutide events/participants	Comparator events/participants	Mantel-Haenszel odds ratio (95% confidence interval)	Wald z	p-value
Reported alopecia or hair loss	9	191/11,564	36/9,799	2.92 (1.99-4.27)	5.52	<0.001
Any injection-site reaction	11	156/5,066	114/3,483	0.86 (0.67-1.11)	-1.14	0.256

Reported Alopecia or Hair Loss

Across nine comparisons, alopecia or hair loss was reported in 191/11,564 semaglutide recipients (1.65%) and 36/9,799 comparators (0.37%). The pooled OR was 2.92 (95% CI: 1.99-4.27; z = 5.52; p < 0.001) (Table 2). At the observed comparator risk of 3.67 per 1,000, the model-based difference was 6.97 additional reports per 1,000 participants (95% CI: 3.62-11.82). This absolute estimate reflects the included trials’ comparator risk, which was strongly influenced by the cardiovascular outcomes trial reported by Lincoff et al. [16]. Figure 2 presents the study-specific and pooled estimates for reported alopecia or hair loss.

**Figure 2:**
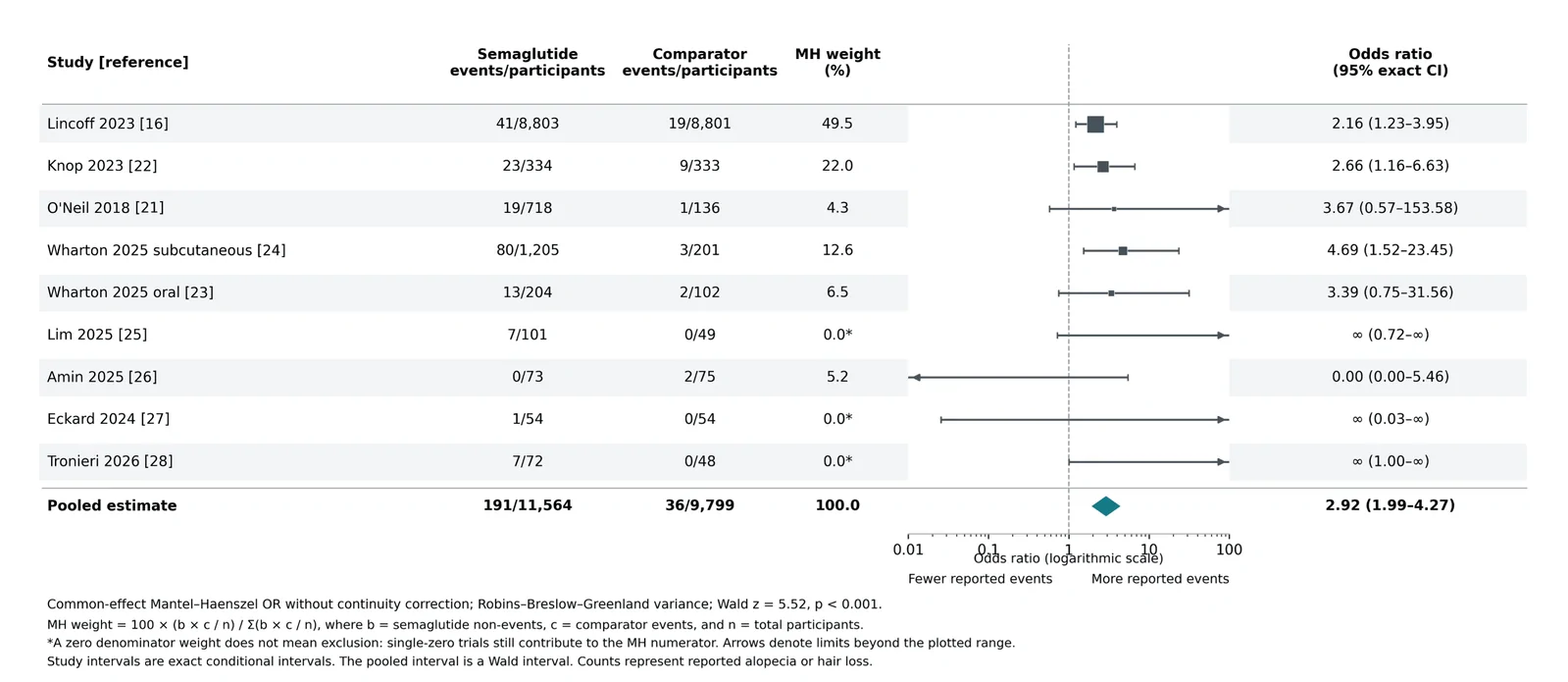
Reported alopecia or hair loss in nine randomized comparisons Squares represent study-specific conditional odds ratios, and horizontal lines show equal-tailed exact 95% confidence intervals. The diamond represents the pooled common-effect Mantel-Haenszel estimate without continuity correction. Square sizes and the weight column show conventional Mantel-Haenszel denominator weights; a study with no comparator events can have zero displayed weight while contributing to the pooled numerator. Arrows indicate estimates or confidence limits beyond the displayed range; ∞ denotes an unbounded estimate or confidence limit. Counts are participants reporting alopecia or hair loss divided by the total participants in the source-specific analysis population. Study labels identify the corresponding trial publications [16,21-28]; linked trial records and safety reports also informed extraction. The restricted adverse-event ascertainment in the trial reported by Lincoff et al. [16] was examined in sensitivity analysis. OR: odds ratio, CI: confidence interval, MH: Mantel-Haenszel, n/N: participants reporting alopecia or hair loss/total participants in the analysis population, HIV: human immunodeficiency virus

Any Injection-Site Reaction

Eleven informative comparisons reported injection-site reactions in 156/5,066 semaglutide recipients (3.08%) and 114/3,483 comparators (3.27%). The pooled OR was 0.86 (95% CI: 0.67-1.11; z = -1.14; p = 0.256) (Table 2). At the observed comparator risk of 32.73 per 1,000, the model-based difference was 4.33 fewer reports per 1,000 participants (95% CI: 10.52 fewer to 3.53 more). The CI was compatible with lower or higher odds of reported reactions. The kidney-transplant-candidacy trial reported 0/7 versus 0/8 participants and contributed descriptive evidence, but no relative-effect information [35]. Figure 3 presents all 12 primary injection-site comparisons and the pooled estimate from the 11 informative trials.

**Figure 3:**
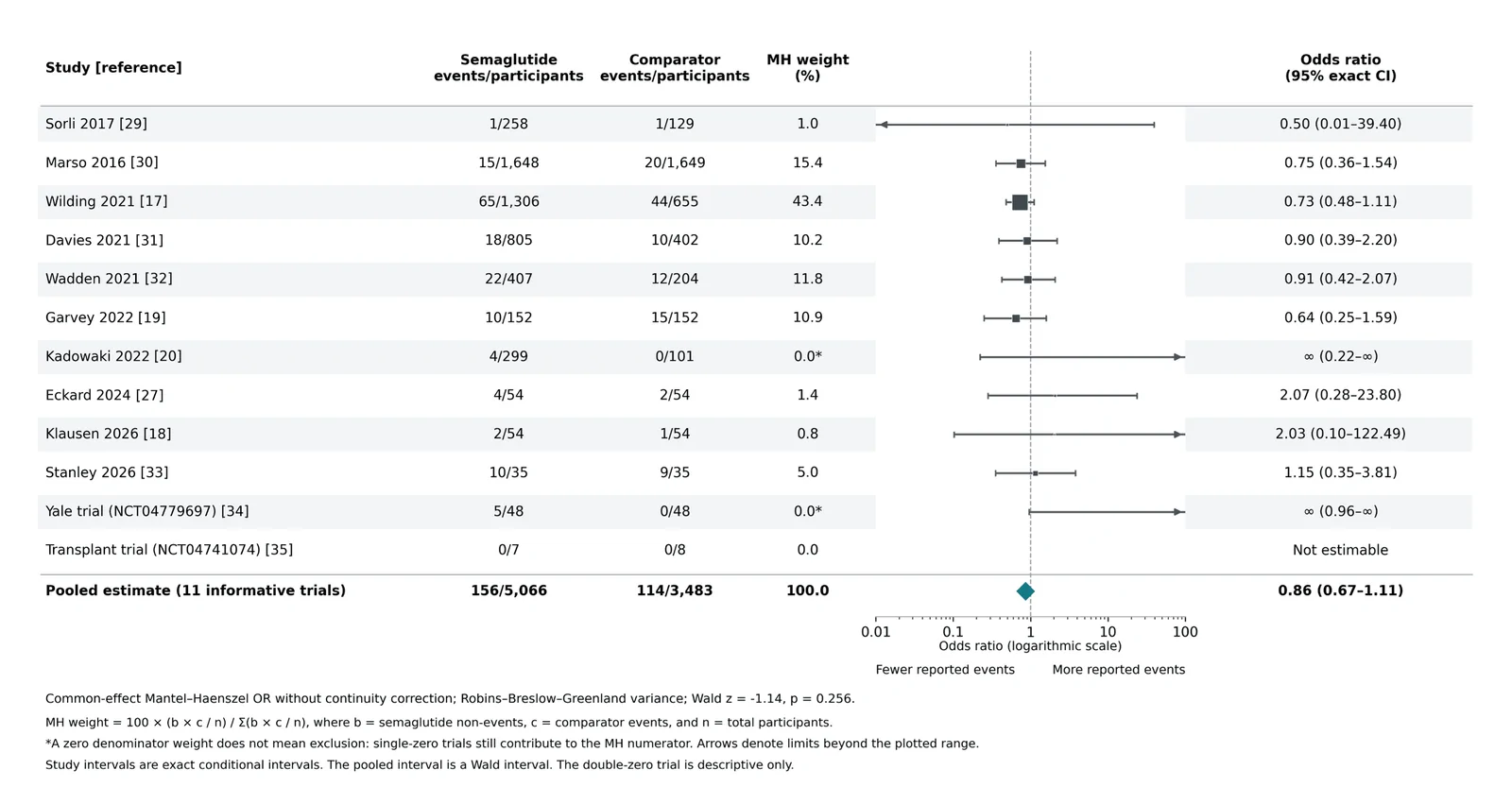
Injection-site reactions in 12 randomized trials Squares represent study-specific conditional odds ratios, and horizontal lines show equal-tailed exact 95% confidence intervals. The diamond represents the pooled common-effect Mantel-Haenszel estimate without continuity correction. Square sizes and the weight column show conventional Mantel-Haenszel denominator weights; a study with no comparator events can have zero displayed weight while contributing to the pooled numerator. Arrows indicate estimates or confidence limits beyond the displayed range; ∞ denotes an unbounded estimate or confidence limit. Counts are participants with at least one source-defined injection-site reaction divided by the total participants in the source-specific analysis population. Eleven trials contributed to the relative-effect estimate; the trial with zero events in both groups was retained descriptively and excluded from the pooled counts. Data were obtained from the cited trial publications, registry results, and linked safety reports [17-20,27,29-35]. The pooled effect was tested using a two-sided Wald z test with Robins-Breslow-Greenland variance: z = -1.14, p = 0.256. The Tarone-adjusted Breslow-Day homogeneity statistic was χ² = 11.05 with 10 degrees of freedom, p = 0.354. NCT identifiers denote ClinicalTrials.gov registration numbers.

Sensitivity Analyses and Other Cutaneous Outcomes

Excluding the trial reported by Lincoff et al. [16] yielded an alopecia OR of 3.66 (95% CI: 2.13-6.27; z = 4.71; p < 0.001; eight comparisons). Excluding the additional phase I registry-only injection-site comparison yielded an OR of 0.82 (95% CI: 0.64-1.06; z = -1.48; p = 0.139; 10 informative comparisons). Excluding the trials by Wilding et al. [17] and Klausen et al. [18], supported by indexed primary-table text, yielded an injection-site OR of 0.95 (95% CI: 0.69-1.33; z = -0.28; p = 0.781; nine informative comparisons). Excluding all three yielded an OR of 0.88 (95% CI: 0.63-1.24; z = -0.72; p = 0.474; eight informative comparisons). Leave-one-out analyses preserved the alopecia association and injection-site CIs spanning one. The exclusion and leave-one-out analyses used the same Mantel-Haenszel model, variance, and two-sided Wald test as Table 2.

The random-effects sensitivity analysis yielded an OR of 2.83 for reported alopecia or hair loss (95% profile-likelihood CI: 1.97-5.28; p < 0.001; nine comparisons) and an OR of 0.86 for injection-site reactions (95% profile-likelihood CI: 0.67-1.23; p = 0.284; 11 comparisons). The maximum-likelihood estimate of τ² was zero for both outcomes, although the profile intervals allowed uncertainty in between-trial variation. These results preserved the direction of the reported hair-loss association and the uncertainty surrounding injection-site reactions.

The cumulative 104-week results of the bimagrumab-semaglutide trial (ClinicalTrials.gov identifier NCT05616013) and the overall-trial results of the diet-adjunct trial (NCT04854083) were excluded from randomized-period comparisons because their observation windows extended beyond identifiable randomized exposure. In the 7.2 mg weight-management trial reported by Wharton et al. [24], dysesthesia-related events were reported in 21.6% of participants receiving semaglutide 7.2 mg and 0.3% receiving placebo; most events in the 7.2 mg group were mild to moderate [2]. These source-reported percentages were summarized descriptively.

Certainty of Evidence

Reported alopecia or hair loss evidence was rated low certainty after downgrading for risk of bias and publication/non-reporting bias. Injection-site evidence was rated very low certainty after an additional downgrade for imprecision. Inconsistency and indirectness warranted no additional downgrading for the broad adult trial-population question. Table 3 presents the absolute effects and certainty ratings.

**Table 3:** Absolute effects and certainty of evidence Absolute differences were calculated from the pooled Mantel-Haenszel odds ratios at the observed pooled comparator risk, held fixed when transforming confidence limits. They describe reported events across differing observation periods and are not annual incidence rates or individual risk predictions. The hair-loss comparator risk is strongly influenced by SELECT’s restricted nonserious adverse-event ascertainment. These are not separately pooled risk differences; relative-effect tests appear in Table 2. Certainty was assessed using the Grading of Recommendations Assessment, Development and Evaluation approach. Both outcomes were downgraded one level for risk of bias: restricted ascertainment and incomplete safety observation for reported alopecia or hair loss, and incomplete follow-up and exposure-related observation for injection-site reactions. Both were downgraded one level for suspected publication/non-reporting bias arising from unavailable eligible outcome results, separately from limitations in included results. SELECT’s restricted ascertainment was counted only under risk of bias. Injection-site reactions were additionally downgraded one level for imprecision because the interval included reduced and increased odds (OR: 0.67-1.11). No additional downgrade was applied for inconsistency or indirectness. The 15 participants in the double-zero injection-site trial were retained descriptively and excluded from relative-effect and transformed absolute-effect totals. Participant totals overlap across outcomes.

Outcome	Participants (informative comparisons)	Comparator risk per 1,000	Absolute difference per 1,000 (95% confidence interval)	Certainty of evidence
Reported alopecia or hair loss	21,363 (9)	3.67	6.97 more (3.62-11.82 more)	Low
Any injection-site reaction	8,549 (11)	32.73	4.33 fewer (10.52 fewer to 3.53 more)	Very low

Active-Comparator and Design-Specific Evidence

Active-comparator results were kept separate because treatments, injection frequency, devices, and comparator exposure differed. The randomized-withdrawal comparison addressed continuation after semaglutide exposure, while the regional double-zero comparison represented a prespecified subset rather than the whole parent trial. These data were not combined with the primary placebo/common-control estimates. Table 4 presents the directly reported counts, including 11 active-comparator outcome rows and two design-specific comparisons [21,36-43].

**Table 4:** Active-comparator and design-specific results retained separately Counts are participants with at least one source-defined outcome divided by the reported analysis denominator. The table contains 13 outcome rows from 12 distinct trials; NCT02453711 also contributes to the primary alopecia analysis, leaving 11 additional trial identities. Different outcome rows from the same trial are not independent studies. No pooled effect or hypothesis test is presented because these comparisons were retained descriptively across different treatments or estimands. A numerical zero indicates a directly reported zero or an exhaustive source statement accounting for all affected participants, not absence from a thresholded table. Sources: NCT02453711 [21]; NCT01930188, NCT01885208, NCT02128932, and NCT02648204 [36]; NCT05822830 [37]; NCT04074161 [38]; NCT03987919 [39]; NCT02207374 [40]; NCT02254291 [41]; NCT03548987 [42]; and NCT04251156 [43] The regional comparison is counted once under its parent trial and contributes no relative odds ratio. mg: milligram

ClinicalTrials.gov identifier	Outcome	Semaglutide events/participants	Comparator and context	Comparator events/participants
NCT02453711	Alopecia or hair loss	19/718	Liraglutide 3 mg/day	3/103
NCT05822830	Alopecia or hair loss	23/376	Tirzepatide maximum tolerated dose	31/374
NCT01930188	Injection-site reaction	3/818	Sitagliptin 100 mg/day; matching weekly injection	2/407
NCT01885208	Injection-site reaction	5/404	Extended-release exenatide 2 mg/week	89/405
NCT02128932	Injection-site reaction	5/722	Insulin glargine daily	6/360
NCT02648204	Injection-site reaction	10/601	Dulaglutide 0.75/1.5 mg/week	12/598
NCT04074161	Injection-site reaction	0/126	Liraglutide 3 mg/day	14/127
NCT03987919	Injection-site reaction	1/469	Tirzepatide 5/10/15 mg/week	43/1,409
NCT05822830	Injection-site reaction	1/376	Tirzepatide 10/15 mg/week	32/374
NCT02207374	Injection-site reaction	2/480	Additional oral antidiabetic drug	0/120
NCT02254291	Injection-site reaction	1/205	Sitagliptin	0/103
NCT03548987	Injection-site reaction	14/534	Switch to placebo after semaglutide run-in	6/268
NCT04251156	Injection-site reaction	0/195	Placebo; prespecified China subgroup	0/105

Outcome-level assessments for the primary placebo/common-control evidence and the separately retained active-comparator and design-specific comparisons used the Cochrane Risk of Bias 2 (RoB 2) tool for the effect of assignment to intervention [9].

**Figure 4:**
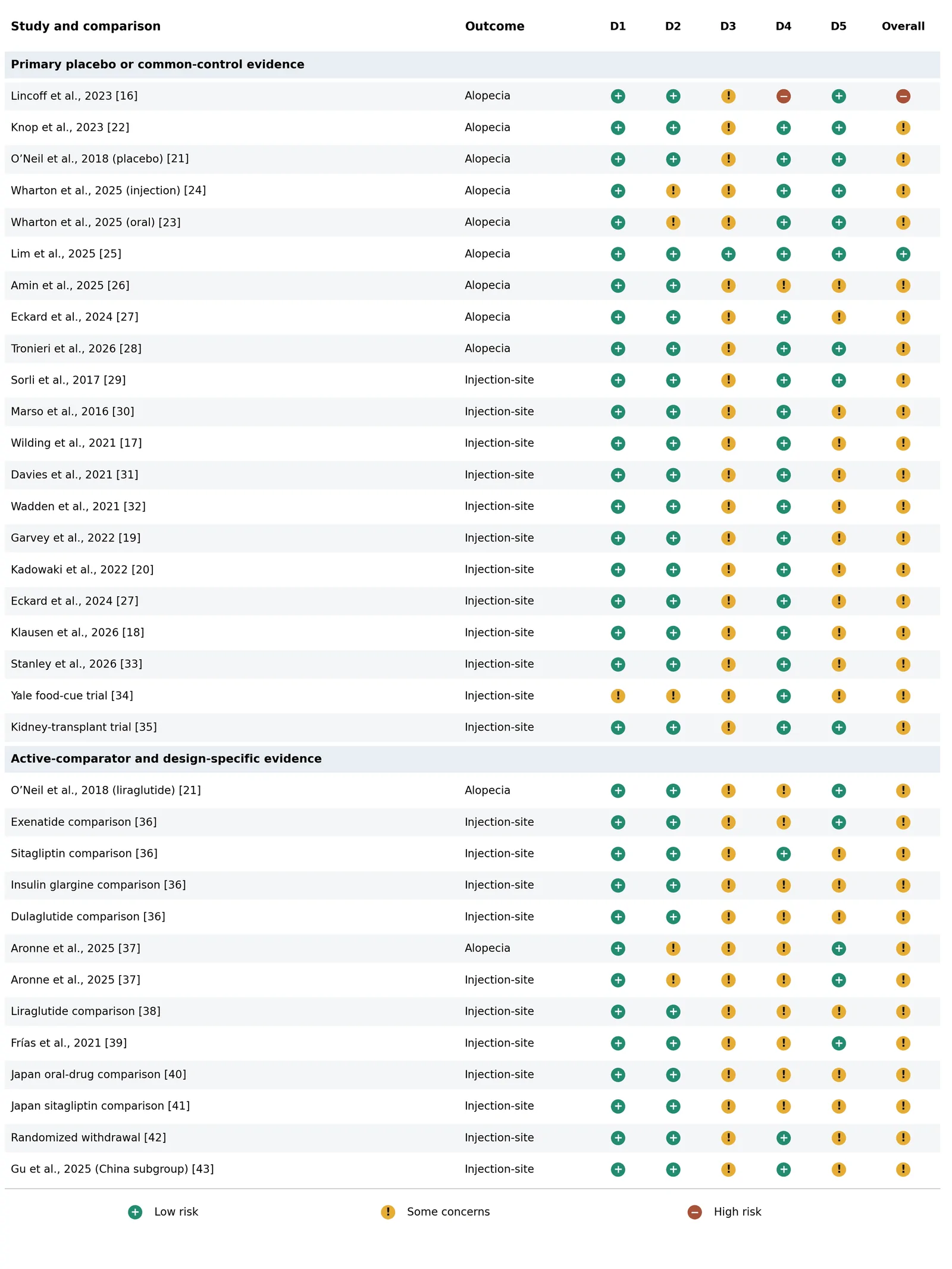
Outcome-specific risk of bias in the included randomized comparisons Assessments used the Cochrane Risk of Bias 2 tool. Domain 1 (D1): randomization process, domain 2 (D2): deviations from intended interventions, domain 3 (D3): missing outcome data, domain 4 (D4): measurement of the outcome, domain 5 (D5): selection of the reported result. Green (+) indicates low risk, amber (!) some concerns, and red (-) high risk. Injection-site denotes the source-defined participant-level injection-site-reaction outcome. The figure contains 34 assessments from 31 trials: 21 primary-evidence assessments and 13 separately retained assessments. The trial with zero events in both groups is included. Restricted adverse-event collection explains the high-risk alopecia judgment for Lincoff et al. [16]; other concerns involved incomplete follow-up, exposure-related observation, unmasked outcome ascertainment, or uncertain prespecification. Sources: trial reports and their linked registrations, protocols, and analysis plans [16-43]

Discussion

Principal Findings

Semaglutide was associated with higher odds of reported alopecia or hair loss than placebo or common control, with a pooled odds ratio of 2.92 (95% CI: 1.99-4.27). The association persisted after exclusion of the large cardiovascular outcomes trial with restricted adverse-event collection. For composite injection-site reactions, the pooled odds ratio was 0.86 (95% CI: 0.67-1.11), leaving the comparative effect uncertain. The low and very low certainty ratings, respectively, are important to interpretation: the results suggest a hair-loss signal but do not establish its mechanism or demonstrate equivalent injection-site safety.

Alopecia in the Context of Previous Evidence

The present findings complement the earlier interventional synthesis by Cheng and Chang [44] and the spontaneous-report analysis by Godfrey et al. [4]. These approaches address different questions. Randomized comparisons estimate differences in reported events between treatment groups, whereas disproportionality analyses identify events reported more often than expected within a safety database. Spontaneous reporting lacks a reliable exposed-population denominator and cannot establish incidence. Tang et al. also investigated hair loss through a target trial emulation in adults with type 2 diabetes [5]. Its observational comparisons support further investigation, although differences in treatment selection and residual confounding distinguish that evidence from randomized trials.

Regulatory information provides additional context. Hair loss is reported in semaglutide weight-management studies, and European product information describes higher frequencies among participants achieving greater weight loss [1,2]. This pattern is compatible with a contribution from physiological changes accompanying weight reduction, but it does not demonstrate mediation or exclude a direct drug effect. The present aggregate data concern reported alopecia or hair loss rather than a consistently confirmed dermatologic diagnosis. Limited information on onset, hair-loss subtype, concurrent illness, nutritional status, and recovery prevents a reliable distinction among potential mechanisms. Although recovery during continued treatment is described in some regulatory datasets [2], the pooled results cannot predict reversibility for an individual patient.

Absolute risk also requires care. At the observed comparator risk, the pooled estimate corresponded to approximately seven additional reports per 1,000 participants. This is a model-based difference across the included follow-up periods, not an annual incidence rate. The comparator risk was strongly influenced by the cardiovascular outcomes trial reported by Lincoff et al., which restricted collection of nonserious adverse events [16]. Excluding that trial preserved the direction of the relative association, but this sensitivity analysis cannot eliminate bias from missing or incompletely ascertained events in the remaining studies. Absolute estimates should therefore be applied in the context of the treatment population and reporting procedures.

Injection-Site Reactions

The uncertain injection-site estimate differs from the class-wide analysis by Taj et al., which combined 14 randomized trials and reported a risk ratio of 3.55 (95% CI: 2.35-5.36) [3]. Their synthesis included different glucagon-like peptide-1 receptor agonists and comparator regimens. The present analysis was specific to semaglutide and required a source-defined participant-level injection-site category. It estimated odds ratios rather than risk ratios, further limiting direct numerical comparison. Differences in drugs, comparators, definitions, and exposure provide plausible explanations for the divergent estimates. Neither synthesis establishes a direct comparative ranking of semaglutide and other agents.

Local-reaction risk depends partly on the injection regimen. Devices, frequency, placebo injections, and double-dummy procedures influence opportunities for reactions in both groups. Composite outcomes may also encompass different combinations of erythema, swelling, pruritus, and other local symptoms. Treating each trial’s composite as a single participant-level outcome avoids counting several reactions in the same person as separate affected participants but does not make the definitions identical. Excluding the additional phase I registry-only trial left the comparative effect uncertain. The confidence interval spanning no difference should not be interpreted as proof of equivalence, and these local-reaction findings do not describe systemic hypersensitivity or overall dermatologic safety.

Clinical Interpretation

The hair-loss signal should be considered alongside semaglutide’s demonstrated weight-management and cardiovascular benefits in appropriate populations [16,17]. The present findings support discussing possible hair loss during treatment reviews, without implying that all new shedding is caused directly by semaglutide or that treatment should routinely be discontinued. Clinical assessment should consider the pattern and timing of hair loss, weight trajectory, concurrent conditions, and other potential contributors. Treatment decisions remain an individualized assessment of benefits, symptom burden, and patient preferences. For injection-site symptoms, the principal message is uncertainty about the comparative effect rather than a protective effect of semaglutide.

Strengths

Linking publications, registrations, and safety reports at the trial level reduced the risk of treating companion reports as independent studies. Participant-level counts, single use of shared controls, separate handling of active comparators, and explicit differentiation between zero events and unreported outcomes supported interpretable comparisons. These features are particularly relevant to harms reviews, in which uncommon events may be omitted from frequency-thresholded tables [8,12].

Limitations

Several limitations remain. Bibliographic searching covered PubMed and Europe PubMed Central rather than every database in the registered plan. Public registers and regulatory sources broadened coverage but cannot ensure that all eligible evidence was identified. The post hoc direct-count requirement narrowed the evidence available for pooling and may have selected trials according to outcome availability. Complete extracted counts are not synonymous with complete event ascertainment: reporting thresholds, restricted safety collection, and missing outcome tables can bias a synthesis even when every included numerator and denominator is known [8,10]. The pooled estimates therefore describe outcome-specific available evidence, not the cutaneous experience of all identified trials. Of 162 eligible trials with an empirical results source, 31 contributed usable data for at least one primary outcome. The absence of usable outcome data in the remaining 131 trials does not imply zero events and may introduce bias related to outcome availability.

Trials differed in indication, formulation, dose, observation period, and adverse-event ascertainment. The random-effects sensitivity analysis preserved the main interpretation, but its zero estimate of between-trial variance cannot exclude clinically important heterogeneity when events are sparse. Neither model corrects selective non-reporting or incomplete ascertainment. The pooled comparisons involved adults, limiting direct application to pediatric populations or unstudied regimens.

Research Priorities

Future trials should report cutaneous outcomes with participant denominators and explicit zero counts, independently of frequency thresholds. Standardized reporting of alopecia subtype, onset, severity, weight change, treatment interruption, and recovery would help distinguish mechanisms and quantify clinically relevant risk. Injection-site reporting should define the composite, its individual components, and the injection exposure. These improvements would strengthen risk communication and future comparative safety analyses.

## Conclusions

Semaglutide may increase reports of alopecia or hair loss compared with placebo or common control, based on low-certainty evidence. Its comparative effect on composite injection-site reactions remains uncertain, with very-low-certainty evidence. Interpretation should account for variation in adverse-event reporting and ascertainment across trials. These findings support clinical awareness of possible hair loss during treatment, with decisions guided by individual benefits and harms. Consistent participant-level reporting of cutaneous events, including their timing, severity, clinical features, and reversibility, would strengthen the evidence and improve risk communication.
